# Correction: Flight delay prediction: Evaluating machine learning algorithms for enhanced accuracy

**DOI:** 10.1371/journal.pone.0353323

**Published:** 2026-07-07

**Authors:** The PLOS One Staff

## Notice of Republication

This article was republished on June 17, 2026, to correct an error in the author list. Samira Dhafir N. AlShahrani was erroneously displayed as Dhafir N. AlShahrani. The correct citation is: AlBassam SAA, AlShahrani SDN (2025) Flight delay prediction: Evaluating machine learning algorithms for enhanced accuracy. PLoS One 20(12): e0335141. https://doi.org/10.1371/journal.pone.0335141. The publisher apologizes for the error. Please download this article again to view the correct version. The originally published, uncorrected article and the republished, corrected articles are provided here for reference.

## Supporting information

S1 File Originally published, uncorrected article. (PDF)

S2 File Republished, corrected article. (PDF)
