# Supplementary material for: Correction: Flight delay prediction: Evaluating machine learning algorithms for enhanced accuracy
Source: PLoS One. 2026 Jul 7;21(7):e0353323. doi: 10.1371/journal.pone.0353323 (PMC13340784; doi:10.1371/journal.pone.0353323)
Supplement: S2 File — (PDF) [file pone.0353323.s002.pdf]

PLOS One

RESEARCH ARTICLE

Flight delay prediction: Evaluating machine learning algorithms for enhanced accuracy

Sarah Ahmed A. AlBassam*, **Samira Dhafir N. AlShahrani**

Department of Information Systems, College of Computer and Information Sciences, King Saud University, Riyadh, Saudi Arabia

* salbassam@ksu.edu.sa

Abstract

Flight delays pose substantial operational and economic challenges for airlines, directly affecting scheduling efficiency, resource allocation, and passenger satisfaction. Accurate prediction of arrival delays is therefore critical for optimizing airline operations and enhancing customer experience. This study systematically evaluates the predictive performance of six machine learning classifiers—Decision Tree, Random Forest, Support Vector Classifier (SVC), Logistic Regression, K-Nearest Neighbors (KNN), and Naive Bayes—on a comprehensive flight dataset, with particular attention to the challenges posed by class imbalance. To mitigate skewed class distributions, resampling techniques including Random Oversampling, Synthetic Minority Oversampling Technique (SMOTE), and Adaptive Synthetic Sampling (ADASYN) were applied to the training data. Model performance was rigorously assessed using stratified 10-fold cross-validation and further validated on a hold-out test set, employing multiple evaluation metrics: Accuracy, F1-score, Matthews Correlation Coefficient (MCC), and ROC-AUC. The results demonstrate that Random Forest combined with Random Oversampling and Decision Tree combined with SMOTE both achieved the highest predictive performance (accuracy 0.90, F1-score 0.90, MCC 0.73, ROC-AUC 0.87. Notably, simpler models such as Naive Bayes exhibited competitive results under balanced conditions, underscoring the continued relevance of probabilistic classifiers in certain operational contexts. These findings highlight the critical role of resampling strategies and rigorous cross-validation in developing reliable, high-performing predictive models for imbalanced flight delay datasets, offering actionable insights for both airline operations and data-driven decision-making.

Check for updates

OPEN ACCESS

Citation: AlBassam SAA, AlShahrani SDN (2025) Flight delay prediction: Evaluating machine learning algorithms for enhanced accuracy. PLoS One 20(12): e0335141. https://doi.org/10.1371/journal.pone.0335141

Editor: Nazanin Tajik, Mississippi State University, UNITED STATES OF AMERICA

Received: February 2, 2025

Accepted: October 7, 2025

Published: December 8, 2025

Copyright: © 2025 AlBassam, AlShahrani. This is an open access article distributed under the terms of the Creative Commons Attribution License, which permits unrestricted use, distribution, and reproduction in any medium, provided the original author and source are credited.

Data availability statement: The data that support the findings of this study are openly available at https://www.kaggle.com/datasets/robikscube/flight-delay-dataset-20182022.

Funding: This research was supported by the Ongoing Research Funding program (ORF-2025-1313), King Saud University, Riyadh,

1. Introduction

Air Transport has become a major factor in global economic growth, allowing for more efficient communication and travel, leading to greater access to goods and services, as well as increased economic opportunities. Annually, approximately 2 billion

Saudi Arabia. The funders had no role in study design, data collection and analysis, decision to publish, or preparation of the manuscript.

Competing interests: The authors have declared that no competing interests exist.

passengers utilize air travel, representing 40% of international tourist movement, demonstrating its crucial role in fostering global connections and tourism growth [1]. This has had a positive impact on economic growth, job creation, and poverty reduction in many countries because air transport creates jobs, facilitates trade, enables tourism, and supports sustainable development around the world [2]. Over the past years, the number of flights in the airline industry has evolved significantly. According to the International Air Transport Association (IATA) in 2019 the airline industry worldwide flew approximately 102,465 flights per day. The demand for air transport has increased by 45% over the last decade, creating a large market for commercial airlines, airports, and other aviation-related businesses. In 2023, all major markets experienced a significant increase in domestic travel demand, with total domestic traffic surpassing the 2019 record. International travel also showed consistent growth globally. As 2024 commenced, the industry's growth remained robust, despite facing economic and geopolitical challenges impacting both airlines and consumers [3]. Therefore, the risk of flight delays has also increased accordingly. It has also led to the development of new technologies: such as aircraft and air traffic control systems. Significantly, reliable flight delay prediction is critical for the air transport industry.

Machine learning methods have been proven to be accurate in predicting flight delays. This study inspects different machine learning algorithms used for flight delay prediction. The motivation for this study comes from the need to improve customer satisfaction because flight delays can be frustrating for travelers, leading to missed connections, lost luggage, and other inconveniences [4]. By accurately predicting flight delays, airlines can proactively notify passengers and offer alternative travel to increase operational efficiency. Meaning flight delays can also be costly for airlines, leading to wasted time, fuel, and other resources. Therefore, in a highly competitive industry, airlines that can consistently deliver on-time flights are more likely to attract and retain customers. By leveraging machine learning to predict and mitigate delays, airlines can gain a competitive advantage over their peers [4].

2. Related work

Several studies on flight delays have been conducted. Researchers have applied machine learning algorithms to predict flight delays effectively. Kalliguddi and Leboulluec [5] developed a flight delay prediction system using multiple linear regression, decision trees, and random forest on over 1 million U.S. domestic flights. The random forest model achieved the best performance with an R-squared (R^2) of 0.94 and RMSE of 12.5 minutes, outperforming other models. The findings also highlighted the key factors contributing to departure delays, including late aircraft arrival, carrier delays, weather conditions, and National Air System (NAS) delays. Another study was conducted by Xu et al. [6] with a focus on analyzing the factors contributing to flight delays and developing a predictive model using statistical analysis and machine learning techniques. The authors analyzed the impact of relevant variables from the dataset, such as temperature, previous delay rate, month, and weekday. As a result, the highest accuracy achieved was 84.5% using Gradient Boosting classifier. However, neither study has resolved the issue of an imbalanced dataset.

In a study by Meel et al. [7], five regression algorithms were evaluated using 2015 U.S. domestic flight data from the Bureau of Transportation Statistics. Models were trained separately for departure and arrival delays. The Random Forest Regressor outperformed others in both cases, achieving the lowest mean square error (MSE) of 2261.8 for departure delays and 3019.3 for arrival delays. Imbalanced dataset issue was not addressed in their work. Moreover, Atlıoğlu et al. [8] compared the performance of eleven supervised learning algorithms in predicting flight delay. The dataset used was provided by local airline company. The findings revealed that classification and regression trees (CART) and K-nearest neighbors (KNN) achieved the highest performance at 0.816 and 0.807 F-Scores respectively. However, they did not employ feature enhancement and instead relied only on the same data acquired from the airline operations. Moreover, they did not utilize any type of data balancing techniques.

Tijil et al. [9] compared the performance of three machine learning algorithms in predicting flight delays using data from the Bureau of Transportation Statistics (BTS). The algorithms applied were Random Forest, Support Vector Machine (SVM), and Logistic Regression. The findings revealed that SVM outperformed the other two algorithms with 100% accuracy. This high level of accuracy might be attributed to the lack of addressing imbalanced data. Chen and Li [10] proposed a flight delay prediction model that integrates multi-label random forest classification with an estimated flight delay propagation model. This approach explicitly considers the propagation of delays across connected flights, leading to improved prediction accuracy. Recognizing the limitations of using all available features, the authors implemented an optimized feature selection technique, demonstrating significant performance gains. Their analysis identified departure delay and late arrival aircraft delay as the most critical factors for robust prediction, with their model achieving an accuracy of 86.72% for arrival delay and 83.05% for departure delay.

Similar to [10], Qu et al [11] work has also focused on delay propagation, where delays in one flight can affect subsequent flights. The authors examined and forecasted flight delays utilizing deep learning models. Two innovative deep learning models were introduced: CBAM-CondenseNet and SimAM-CNN-MLSTM. The research demonstrated that these models can effectively capture both spatial and temporal dependencies in flight data, achieving accuracies of 89.8% and 91.36%, respectively. While the approach effectively captures delay propagation patterns, it does not address the class imbalance issue.

Moreover, Güvercin et al. [12] addressed flight delay prediction by leveraging airport network structure and delay patterns from similar airports. Using graph-based metrics like betweenness centrality and articulation points, the authors proposed Clustered Airport Modeling (CAM) to forecast arrival delays across 305 U.S. airports. Their approach improved forecasting accuracy by up to 55% MAPE over individual models. However, their focus remains on statistical time series modeling. It does not incorporate machine learning or address class imbalance, key gaps targeted in this study.

Deep learning algorithms have also been applied to predict flight delays. Ayaydin and Akçayol [13] applied deep recurrent neural network (DRNN), long-short term memory (LSTM), and Random Forest (RF) models to classify flight delays using a real-world dataset covering 368 global airports. Among the models, LSTM achieved the highest recall (96.50%), while Random Forest led in accuracy (82.21%) and F1-score (96.20%). Although the study applied standard preprocessing and hyperparameter tuning, it did not address feature selection or class imbalance, which are core aspects this work aims to enhance. Bisandu et al. [14] proposed a flight delay prediction model that combines deep recurrent neural network (DRNN) and a social ski driver conditional autoregressive-based (SSDCA-based) deep learning algorithm. The used dataset is from the US Government Bureau of Transportation Statistics (BTS). The model achieved an accuracy of % 93.61% and % 92.52% on dataset1 and dataset2, respectively, demonstrating superior performance compared to other methods. However, its complexity and reliance on metaheuristic tuning may limit real-time deployment or scalability across diverse aviation environments. Furthermore, the data imbalanced issue was not addressed.

Zhou et al. [15] analyzed the factors influencing departure time delay. They have also proposed gated recurrent unit (GRU) model to predict actual flight departure times using operational and scheduling data from Nanjing Lukou Airport. Their findings revealed that the proposed model achieved an RMSE of 0.42 and MAE of 0.3, and outperformed LSTM,

BP, and Random Forest. While the model shows high accuracy, its static nature and reliance on single-airport data limit adaptability to real-time or multi-airport environments. Cai et al. [16] proposed a graph-based deep learning framework, MSTAGCN, to predict flight delays across a multi-airport network using time-evolving graph-structured data. Using domestic flight records from several Chinese airports, the model outperformed baseline approaches such as STGCN and DCRNN in both short and long-term predictions. However, its complexity and need for high-resolution graph data may limit scalability in real-time or low-data settings.

Li et al. [17] developed a two-stage CNN-LSTM-Random Forest model for flight delay prediction, capturing spatial dependencies via CNN, temporal weather effects via LSTM, and fusing both with extrinsic flight features in a Random Forest classifier. Using U.S. domestic flight data, the model achieved 92.39% accuracy. However, its multi-stage design may add complexity for real-time implementation. Deng et al. [18] proposed a modular neural network architecture called CC-MIDNN for predicting aircraft estimated arrival time (EAT), combining k-means clustering, Bayesian optimization, and a deep neural network (DNN) ensemble to train parallel sub-networks. Evaluated on Lisbon Airport data, it reduced MAE by 5.92 minutes over baseline models. However, its multi-step integration process and computational complexity may challenge deployment in real-time operational systems.

Kim and Park [19] focused on long-term predictions of flight delays, which exceed 2 hours. The datasets were collected from three different airports: Incheon International Airport in South Korea (ICN), John F. Kennedy International Airport (JFK), and Chicago Midway International Airport (MDW) in the United States. Several machine learning models, and Long Short-Term Memory (LSTM) were applied to predict flight takeoff delays, achieving up to 85.2% accuracy with the LSTM model at JFK. The imbalance issue was solved using random under sampling technique. This approach discards valuable data and may reduce model robustness. In a recent study, Yuan et al. [20] introduced a hybrid deep learning model (3DF-DSCL) combining 3D-CNN, GCN, and LSTM to predict airport departure delays using multi-attribute data (temporal, spatial, and spatiotemporal). Utilizing flight data from Beijing Capital International Airport, the model achieved a Mean Absolute Error (MAE) of 0.26 minutes, outperforming existing approaches by 14.47%. However, the model's dependence on detailed operational data from a single airport may limit its applicability to broader contexts.

Table 1 presents a summary of previous related works. Generally, machine learning techniques have proved to be effective for predicting flight delays. However, most of the previously mentioned studies lack some important aspects. Either they did not address the imbalanced data issue, or they missed important steps that affect the findings, such as feature selection and feature engineering. Deep learning techniques show potential for flight delay prediction; however, they often require substantial computational resources and large labeled datasets for effective training.

To overcome these limitations, we propose an improved model for predicting flight delays, which includes several techniques to improve the performance of classifiers and thus find the best model to achieve our goal. The key objective of this project is to develop a ML model that can effectively detect small (< 15 mints), medium (≥ 15 and <45 mints) and large (≥45 mints) flight delays. In our study, we considered the issue of imbalanced dataset and applied feature selection and feature engineering techniques to improve the findings.

3. Proposed methodology

In this study we proposed an improved flight delay prediction model. To develop the model six different supervised learning algorithms were investigated and compared to select the best classifier. Several steps of feature engineering, feature selection, data resampling, data scaling and hypermeter optimization were applied to enhance the performance. An overview of the proposed methodology is shown in Fig 1.

3.1. Experiment environment

All experiments were conducted on a MacBook Pro equipped with an Intel Core i7 processor and 16 GB of RAM. The model implementation environment is Google Collaboratory (Colab), a cloud based Jupyter Notebook

Table 1. Summary of related work.

Paper Ref.	ML/DL Algorithms	Performance Measures	Dataset	Feature Selection Methods	Feature Engineering	Sampling Techniques
[5]	multiple linear regression, decision tree, and random forest	R-squared (R^2) 0.94<br>Root Mean Squared Error (RMSE) 12.5	Bureau of Transportation Statistics (BTS)	no	no	No
[6]	Gradient Boosting<br>Logistic Model,<br>K-Near Neighbors<br>Gausian NB,<br>Support Vector Machine,<br>Decision Tree Classifier,<br>Random Forest Classifier	Accuracy 84.5	Chinese Air Traffic Control Authority	yes	yes	No
[7]	Logistic Regression, Decision Tree Regression, Bayesian Ridge, Random Forest Regression, Gradient Boosting Regression	Mean squared error (MSE)<br>Departure 2261.8<br>Arrival 3019.3	the US Bureau of Transport Statistics 2015	Manual feature choice	no	No
[8]	K-Nearest Neighbor (KNN), Support Vector Machine (SVM), Decision Tree (CART),<br>Gaussian Naïve Bayes (GNB), Logistic Regression (LR), Multilayer Perceptron (MLP),<br>Random Forest (RF), Gradient Boosting (GBM),<br>XGBoost (XGB), CatBoost (CB), and LightGBM (LGBM)	F score<br>0.816 and 0.807	Turki airline company	yes	yes	No
[9]	Random Forest,<br>Support Vector Machine SVM,<br>Logistic Regression	f-score, recall, precision, and accuracy 100% scores.	The Bureau of Transportation Statistics (BTS)	no	yes	No
[10]	combined multi-label random forest classification and approximated delay propagation model	Accuracy<br>Arrival delay 86.72<br>Departure delay 83.05	Three databases:<br>Bureau of Transportation Statistics (BTS)<br>Local Climatologica Data (LCD)<br>Aviation<br>System Performance Metrics (ASPM)	recursive feature elimination (RFE) algorithm	yes	SMOTE
[11]	CBAM-CondenseNet<br>SimAM-CNN-MLSTM	Accuracy<br>89.8% and 91.36%, respectively.	China Air Traffic Management Bureau	yes	yes	No
[12]	regression and time series forecasting	Mean Absolute Percentage Error (MAPE) and<br>Mean Absolute Error (MAE)	Flight data from 305 us airports	yes	yes	No
[13]	deep recurrent neural network (RNN), long-short term memory (LSTM), and random forest (RF)	Recall 96.50%.	real data set covering 368 airports across the world	yes	yes	No
[14]	deep recurrent neural network (DRNN)	Accuracy<br>%93.61 dataset1%92.52 dataset2	the US Bureau of Transport Statistics 2019–2020	yes	yes	No
[15]	gated recurrent unit (GRU) model	RMSE of 0.42 and MAE of 0.3	Nanjing Lukou Airport	no	yes	no
[16]	a graph-based deep learning framework	MAE 5.884	Chinees airports	no	yes	no

(Continued)

Table 1. (Continued)

Paper Ref.	ML/DL Algorithms	Performance Measures	Dataset	Feature Selection Methods	Feature Engineering	Sampling Techniques
[17]	Two-stage CNN-LSTM-Random Forest model	Accuracy 92.39%	Bureau of Transport Statistic	no	yes	no
[18]	neural network architecture CC-MIDNN	MAE 5.92	Lisbon Airport (LIS), Portugal	no	yes	no
[19]	Decision Tree, Random Forest, Support Vector Machine, K-nearest neighbors, Logistic Regression, Extreme Gradient Boosting, and Long Short-Term Memory	Accuracy 85.2%	datasets were collected from three different airports	yes	yes	Random under sampling
[20]	hybrid deep learning model (3DF-DSCL)	MAE 0.26	Beijing Capital International Airport	yes	yes	no

https://doi.org/10.1371/journal.pone.0335141.t001

environment that provides seamless access to CPUs, GPUs, and TPUs without any local configuration. Python was used as the programming language, enabling the integration of a wide range of libraries essential for machine learning workflows. Specifically, Scikit-learn, NumPy, Pandas, Matplotlib and Seaborn the libraries were employed.

3.2. Data source

The study leverages the publicly available "Flight Status Prediction" dataset [21] hosted on Kaggle, comprising approximately 4,078,318 records across 61 columns, totaling around 51.94 MB. The dataset spans 2018–2022 and includes comprehensive flight information such as:

- Departure and arrival airports, flight numbers, and airline codes
- Scheduled and actual departure and arrival times
- Arrival and departure delays, cancellations, and associated operational delays (carrier, weather, NAS, security, and late aircraft delays)
- Flight origin, destination, distance, airtime, and elapsed times

The dataset captures a wide variety of domestic and international flights, operated by multiple carriers across different time zones.

3.3. Data pre-processing

Exploratory data analysis (EDA) was conducted to understand the dataset's characteristics and relationships between variables. Key Python libraries (NumPy, Pandas, Matplotlib, Seaborn) were used for data manipulation and visualization. Initial exploration confirmed the dataset had time-series flight information with no missing values. Descriptive statistics were obtained using df.describe() to summarize central tendencies, dispersion, and potential outliers.

3.4. Feature engineering

To enhance model performance and interpretability, feature engineering was applied. This included creating meaningful features from existing data to improve model predictions [22]. Specifically, the "delay_hours" column was binned into three categories as shown in Table 2:

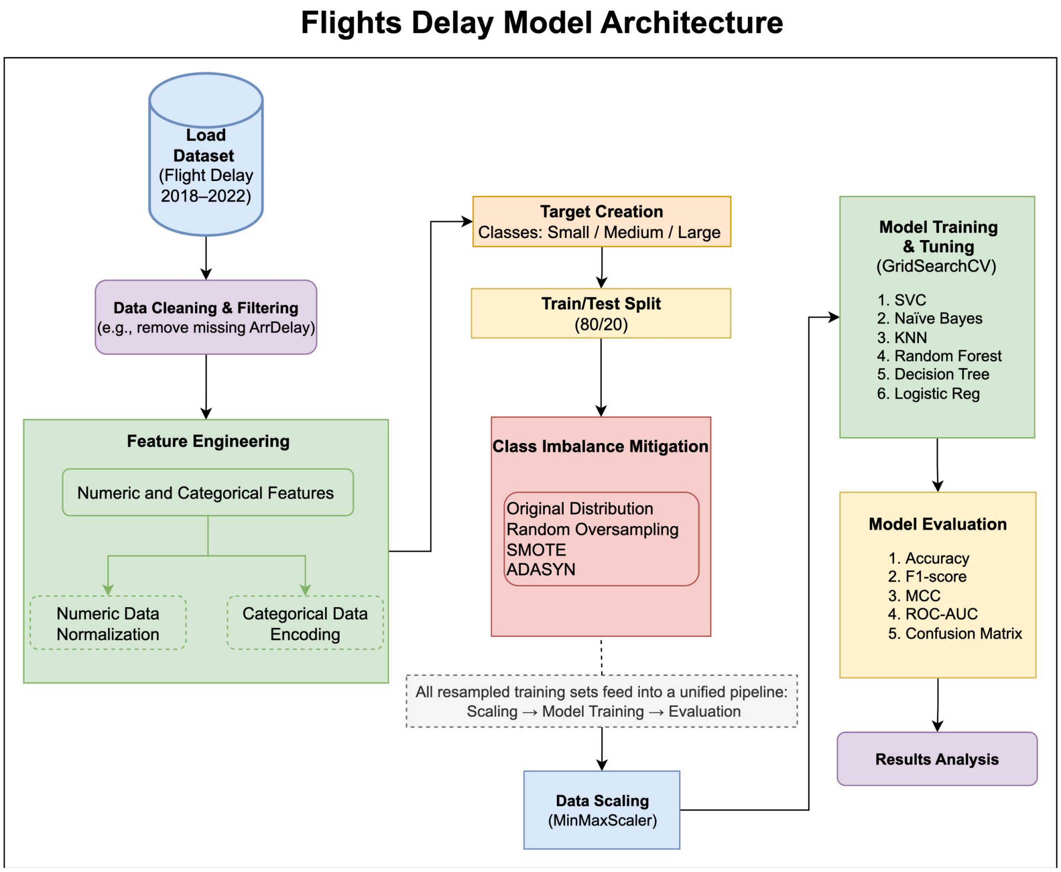

Fig 1. Overview of the study methodology.

https://doi.org/10.1371/journal.pone.0335141.g001

This transformation converts the problem into a multiclass classification task, capturing operationally meaningful delay thresholds. Additional engineered features included time-based aggregations, such as departure hour bins, day of the week, and day of the year, which have been shown in prior studies to influence flight punctuality.

3.5. Feature selection

Feature selection is a crucial step for optimizing model performance. It involves identifying and selecting the most relevant features from the dataset to improve predictive accuracy and reduce overfitting [23]. In this study, we used the SelectK-Best method with K = 25, which selects the top 25 features based on their scoring function. After preprocessing, a total of 25 features were selected based on domain knowledge and correlation analysis.

The features included are: FlightDate, DayOfWeek, Month, Carrier, FlightNumber, OriginAirport, DestAirport, ScheduledDepTime, ScheduledArrTime, Distance, ActualDepTime, ActualArrTime, DepDelay, ArrDelay, TaxiOutTime, TaxiInTime, CarrierDelay, WeatherDelay, NASDelay, SecurityDelay, LateAircraft Delay, PrevFlightDelay, DayOfYear, DepHourBin, DelayCategory.

This curated feature set balances interpretability with predictive capability and ensures that only relevant, non-redundant information is fed into the models.

Table 2. Data binning.

Delay Category	Delay In Minutes
Small	< 15
Medium	15–44
Large	≥ 45

https://doi.org/10.1371/journal.pone.0335141.t002

3.6. Data resampling

Data resampling techniques are used to solve the imbalance data classification issue, where the dataset has an unequal distribution of the instances of each class, causing the classification model to be biased towards the majority class and reducing its overall performance [24]. Flight delay datasets are inherently imbalanced, with On-time flights often dominating the data. To address this, three resampling strategies were employed:

1. Random Oversampling (ROS): Minority classes were randomly duplicated until class balance was achieved.
2. Synthetic Minority Oversampling Technique (SMOTE): Synthetic samples were generated for minority classes using k-nearest neighbors in feature space.
3. Adaptive Synthetic Sampling (ADASYN): Focused on generating synthetic samples for minority class instances that are harder to learn, improving classifier robustness for difficult examples.

These methods aim to mitigate bias toward majority classes and enhance the classifier's ability to detect all delay categories effectively.

3.7. Data splitting

Data splitting is a technique for dividing the dataset into two parts: a training set and a testing set. The training set is used to train the model, and the testing set is used to evaluate the model's performance on unseen data. The data splitting is commonly implemented using the train_test_split function, which allows the allocation of a pre-defined proportion of data for testing purposes. The test_size parameter is set to 0.2, indicating that 20% of the data will be assigned for testing purposes, while the remaining 80% will include the training set. This strategic division serves to ensure an objective evaluation of the model's performance on unseen data, thereby mitigating the risk of overfitting.

3.8. Data scaling

For machine learning algorithms, having feature values that are closer together generally improves both the speed and effectiveness of training. Conversely, when data points or feature values are spread apart, it can take longer for the model to learn, potentially reducing accuracy. To address this, scaling is used to bring data points closer together. Essentially, scaling standardizes the values, making them more comparable. Different scaling techniques, such as Min-Max Scaler, Max Abs Scaler, and Quantile Transformer Scaler, are available to accommodate various classification tasks and enhance the effectiveness of scaling [25,26]. In our model, we implemented the MinMaxScaler method. The MinMaxScaler method helped our model to normalize each feature by scaling it to a specific range, typically between 0 and 1. This can be helpful for machine learning algorithms that are sensitive to the scale of the features by calculating the minimum and maximum values for each feature.

3.9. Hyperparameter tuning

To optimize model performance, hyperparameter tuning was conducted for all classifiers. Hyperparameters define key aspects of each algorithm, such as the number of neighbors in K-Nearest Neighbors, the regularization strength in Logistic Regression, or the depth of trees in Decision Tree and Random Forest classifiers.

Selecting optimal hyperparameters ensures a balance between underfitting and overfitting, improving generalization on unseen data. In this study, a grid search method was applied to each model.

3.10. Model algorithms

This study evaluates six supervised learning algorithms for predicting flight delays, selected to provide a balance of diverse modeling approaches, from linear to non-linear, and from simple to ensemble methods. The rationale for including these specific algorithms is to compare their performance across different modeling strategies and identify the most effective approach for this dataset.

1. Support Vector Classifier (SVC): A supervised learning algorithm that classifies data by finding the optimal decision boundary (hyperplane) between classes. It can handle both linear and non-linear problems using kernel functions and is highly effective in high-dimensional spaces [27].
2. Naïve Bayes (NB): A probabilistic classifier based on feature independence assumptions; it is simple, fast, and performs well with categorical data [27].
3. K-Nearest Neighbors (K-NN): A non-parametric method that classifies instances based on proximity to neighboring points, providing a simple yet flexible baseline [27].
4. Random Forest (RF): An ensemble of decision trees that improves accuracy and robustness, reducing overfitting common in single trees.
5. Decision Tree (DT): A straightforward model that recursively splits data based on information gain, offering interpretability and fast training [28].
6. Logistic Regression (LR): A statistical linear model that estimates the probability of categorical outcomes, serving as a benchmark for comparison with more complex models [27].

3.11. Cross-validation

Cross-validation is a method used to assess a model's effectiveness and evaluate its performance by training it on one subset of the data and testing it on a different, unseen subset. A single evaluation run may not provide a reliable measure of the model's performance. To ensure a thorough assessment and enhance the model's robustness, repeated iterations are preferred. In our study, we employed 10-fold cross-validation. The final performance scores of the models were determined by averaging the results from all ten iterations.

3.12. Evaluation metrics

To evaluate the performance of the classifiers, four key metrics were employed: accuracy, F1-score, Matthews Correlation Coefficient (MCC), and ROC-AUC. These measures collectively provide both an overall performance snapshot and insights into the models' discriminative abilities.

A confusion matrix is used to summarize classification results. It presents true positives (TP), false negatives (FN), true negatives (TN), and false positives (FP) [29].

I. Accuracy is the percentage of correctly identified flights. It calculated as the ratio of correctly identified cases (delay and on time) to the total number of cases.

$$Accuracy = \frac{TP + TN}{TP + TN + FP + FN} \tag{1}$$

II. The F1-score depends on both precision and recall. It is calculated by the following formula.

$$F1 - Score = 2 \times \frac{Precision \times Recall}{Precision + Recall} \tag{2}$$

III. Matthews Correlation Coefficient (MCC) provides a balanced measure even for imbalanced datasets.

$$MCC = \frac{TP \times TN - FP \times FN}{\sqrt{(TP + FP)(TP + FN)(TN + FP)(TN + FN)}} \tag{3}$$

IV. ROC-AUC (Receiver Operating Characteristic – Area Under Curve) measures the ability of the classifier to distinguish between classes, with values closer to 1 indicating better performance.

4. Results

4.1. Models performance across resampling methods

The performance of six machine learning algorithms—Decision Tree (DT), Random Forest (RF), Support Vector Classifier (SVC), Logistic Regression (LR), K-Nearest Neighbors (KNN), and Naive Bayes (NB)—was evaluated across three resampling strategies: Random Oversampling, SMOTE, and ADASYN. The results, summarized in Tables 3–8, indicate that the choice of resampling technique substantially impacts classifier efficacy, particularly under class imbalance conditions.

As shown in Table 3, Random Forest attained its highest accuracy of 0.90 using Random Oversampling, achieving an F1-score of 0.91, MCC of 0.74, and ROC-AUC of 0.87. While SMOTE and ADASYN yielded modestly lower accuracies of 0.87, Random Oversampling enabled the model to more reliably classify both delayed and on-time flights. The confusion matrix in Fig 2 highlights this balanced performance.

Table 4 demonstrates that Decision Tree achieved maximum accuracy (0.90) with SMOTE, accompanied by an F1-score of 0.91, MCC of 0.74, and ROC-AUC of 0.87. Fig 3 illustrates the confusion matrix under SMOTE, indicating a substantial reduction in misclassifications compared to other resampling methods. These results emphasize the capacity of SMOTE to enhance the Decision Tree's handling of minority classes.

Table 5 presents the performance of Naïve Bayes. Using SMOTE resampling. Naïve Bayes demonstrated relatively modest results with an accuracy 0.89, F1-score 0.89, MCC 0.71, ROC-AUC 0.86. Fig 4 highlights the SMOTE-enhanced confusion matrix.

Among the six classifiers, K-Nearest Neighbors (KNN) exhibited the lowest performance as shown in Table 6. Random Oversampler produced the best results (accuracy 0.54, F1-score 0.59, MCC 0.11, ROC-AUC 0.56). Fig 5 confirms the superiority of Random Oversampler, suggesting that KNN performs better with simpler oversampling rather than synthetic data generation.

Table 3. Random forest with resampling technique.

Resampling Technique	Accuracy	F1-score	MCC	ROC-AUC
Random Oversampler	**0.90**	**0.91**	**0.74**	**0.87**
SMOTE	0.87	0.88	0.68	0.87
ADASYN	0.87	0.88	0.68	0.86

https://doi.org/10.1371/journal.pone.0335141.t003

Table 4. Decision tree with resampling technique.

Resampling Technique	Accuracy	F1-score	MCC	ROC-AUC
RandomOversampler	0.89	0.89	0.70	0.85
SMOTE	**0.90**	**0.91**	**0.74**	**0.87**
ADASYN	0.85	0.86	0.65	0.86

https://doi.org/10.1371/journal.pone.0335141.t004

Table 5. NaiveBayes with resampling technique.

Resampling Technique	Accuracy	F1-score	MCC	ROC-AUC
RandomOversampler	0.88	0.89	0.70	0.86
SMOTE	**0.89**	**0.89**	**0.71**	**0.86**
ADASYN	0.87	0.88	0.68	0.86

https://doi.org/10.1371/journal.pone.0335141.t005

Table 6. K-nearest neighbors with resampling technique.

Resampling Technique	Accuracy	F1-score	MCC	ROC-AUC
RandomOversampler	**0.54**	**0.59**	**0.11**	**0.56**
SMOTE	0.51	0.57	0.12	0.57
ADASYN	0.50	0.56	0.10	0.56

https://doi.org/10.1371/journal.pone.0335141.t006

Table 7. Logistic regression with resampling technique.

Resampling Technique	Accuracy	F1-score	MCC	ROC-AUC
RandomOversampler	0.87	0.87	0.67	0.85
SMOTE	0.86	0.87	0.66	0.85
ADASYN	**0.88**	**0.88**	**0.69**	**0.86**

https://doi.org/10.1371/journal.pone.0335141.t007

Table 8. SVC with resampling technique.

Resampling Technique	Accuracy	F1-score	MCC	ROC-AUC
RandomOversampler	0.87	0.88	0.70	0.89
SMOTE	0.88	0.89	0.71	0.87
ADASYN	**0.89**	**0.91**	**0.73**	**0.89**

https://doi.org/10.1371/journal.pone.0335141.t008

Logistic Regression outcomes are summarized in Table 7. Overall, the ADASYN performed better than Random Oversampling and SMOTE, with an accuracy of 0.88 and F1 score of 0.88. Fig 6 displays the confusion matrix with ADASYN.

SVC has also exhibited good performance with ADASYN, it attained an accuracy of 0.89, F1-score of 0.91, MCC of 0.73, and ROC-AUC of 0.89 as shown in Table 8. Both Random Oversampling and SMOTE produced competitive results. Fig 7 presents the ADASYN-enhanced confusion matrix, which illustrates a highly balanced classification across delay categories, indicating that SVC effectively captures complex, non-linear relationships in the flight delay dataset.

Based on the overall performance metrics, Random Forest using Random Oversampling and Decision Tree using SMOTE sampling emerged as the top performers, consistently achieving the highest accuracy and F1-scores. Their ability to correctly classify both positive and negative instances make them suitable choice for the flight delay classification task.

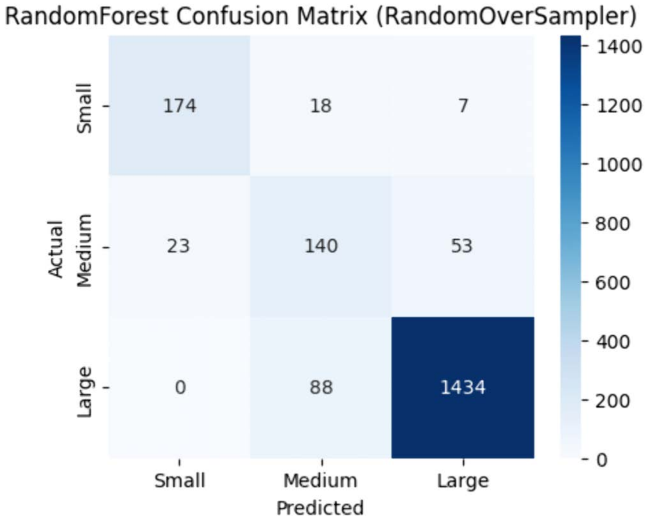

Fig 2. Random forest confusion matrix | best resampler: random oversampler.

https://doi.org/10.1371/journal.pone.0335141.g002

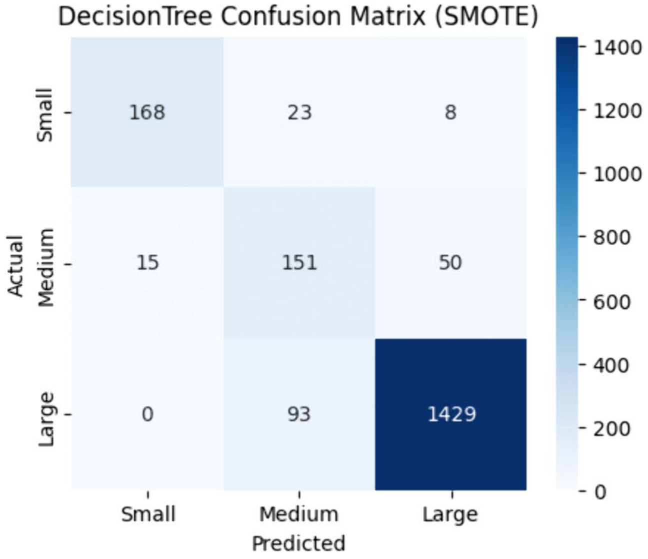

Fig 3. Decision tree confusion matrix | best resampler: SMOTE.

https://doi.org/10.1371/journal.pone.0335141.g003

4.2. Statistical analysis

To evaluate whether the observed differences in predictive performance were statistically significant, two complementary tests were conducted: the paired t-test and the McNemar test. These analyses provide a robust framework for determining whether differences in model performance reflect genuine distinctions or are due to random chance.

Since multiple top-performing models exhibited very similar Accuracy and other evaluation metrics, a direct comparison between the best and worst-performing models was conducted to clearly illustrate the statistical and practical differences in predictive performance. This approach ensures that the analysis highlights the maximum contrast between model

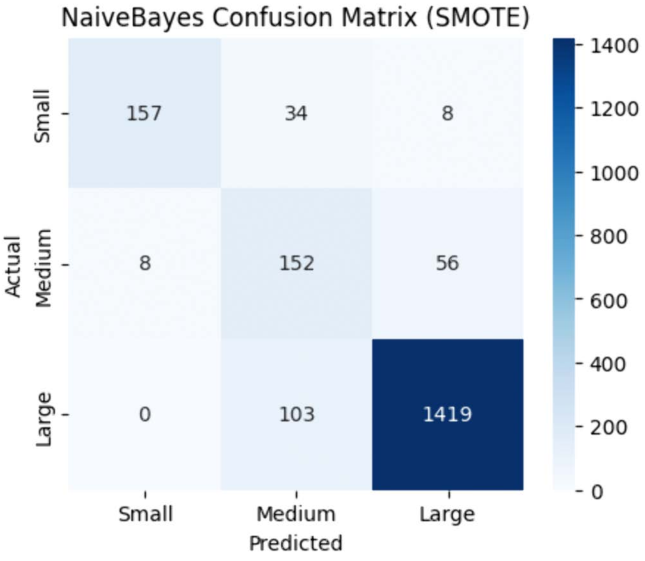

Fig 4. NaiveBayes confusion matrix | best resampler: SMOTE.

https://doi.org/10.1371/journal.pone.0335141.g004

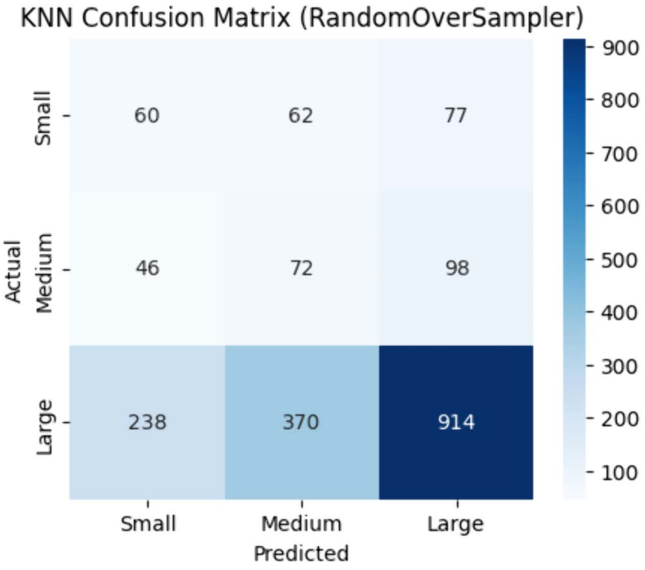

Fig 5. K-nearest neighbors confusion matrix |best resampler: randomoversampler.

https://doi.org/10.1371/journal.pone.0335141.g005

capabilities, providing a more interpretable and informative evaluation. Thus, Random Forest with RandomOverSampler was compared to K-Nearest Neighbors with ADASYN.

5.2.1. Paired t-test. The paired t-test was employed to compare the correctness of predictions for individual flights between the best-performing model (Random Forest with RandomOverSampler) and the worst-performing model (K-Nearest Neighbors with ADASYN). The resulting statistics were:

- Paired t-test between RandomForest and KNN (test set accuracy): $t = 30.9884$, $p < 0.001$

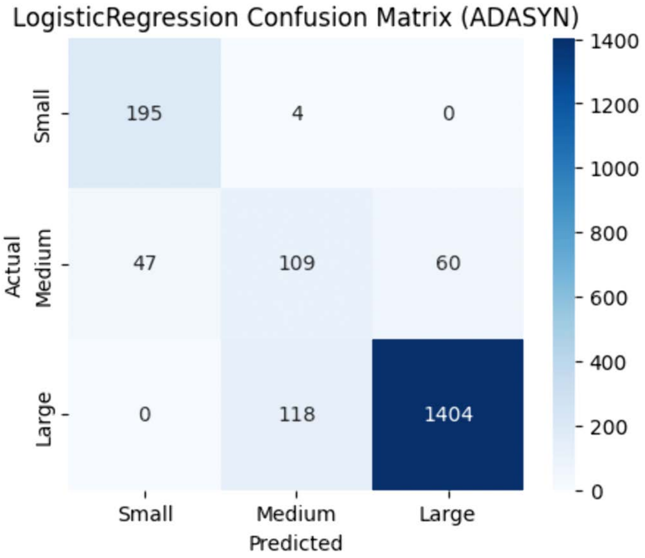

Fig 6. Logistic regression confusion matrix |best resampler: ADASYN.

https://doi.org/10.1371/journal.pone.0335141.g006

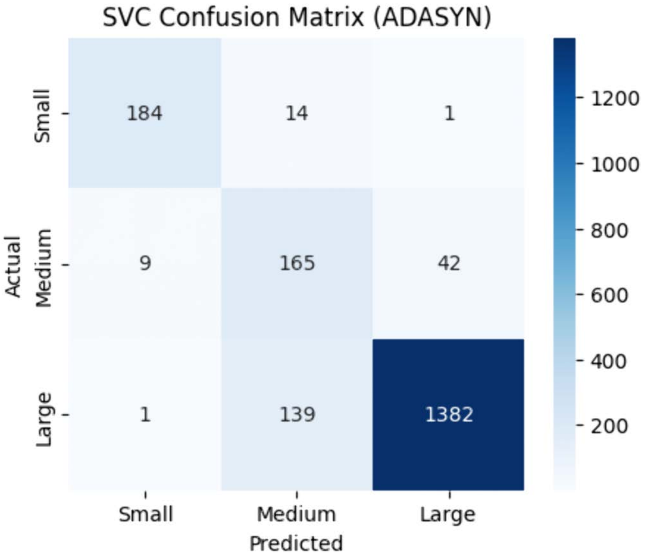

Fig 7. SVC confusion matrix | best resampler: ADASYN.

https://doi.org/10.1371/journal.pone.0335141.g007

The extremely low p-value indicates a highly significant difference in predictive performance, with Random Forest correctly classifying a substantially larger proportion of flights compared to KNN.

5.2.2. McNemar test. The McNemar test was applied to the test set predictions to evaluate whether there was a significant difference in misclassification patterns between the two models. The test produced the following results:

- McNemar test statistic = 78.0, p-value ≈ 9.02×10^{-165}

The very low p-value confirms that Random Forest and KNN exhibit significantly different misclassification patterns. In particular, Random Forest demonstrates superior ability to correctly identify delayed flight instances, whereas KNN struggles with certain delay categories.

5. Discussion

Our goal is to propose an efficient ML flight delay prediction model. So, we investigated six different ML algorithms and applied feature engineering and feature selection to improve the performance of the model. We have also addressed the issue of an imbalanced dataset.

Our findings revealed that Random Forest with Random Oversampler and Decision Tree with SMOTE, outperformed other algorithms for the flight delay prediction task. This aligns with some previous works on flight delay prediction, which found that Random Forest outperformed other algorithms [5,7,19].

Some previous studies [6–8,10–14,19] have recognized the limitations of using all available features and implemented an optimized feature selection technique, demonstrating significant performance gains. Compared to those studies that applied feature selection, the performance of our model outperforms most of the previous works [6–12,19] achieving an accuracy of 0.90 and an F-score of 0.91 for both Random Forest and Decision Tree algorithms. Although the study [14] achieved higher performance measures with an accuracy of 93.61%, it did not address the data imbalance issue. The performance of classifiers is expected to decline after addressing the data imbalance issue. This decline occurred because, prior to balancing, the models were biased toward the majority class and were largely unaffected by misclassifying the minority class. However, after correcting the class distribution, the models classified both classes more equitably, leading to a decrease in performance but a more realistic and balanced approach [30].

None of the previous works cited in this paper has addressed the issue of class imbalance. In contrast to Chen et al [10], the only study that applied the same sampling technique (oversampling) to tackle the problem of imbalanced data, our model achieved better performance, with an accuracy of 90%, while they obtained an accuracy of 86.72%. Therefore, after addressing the class imbalance, our proposed model outperformed the previous studies referenced in this paper.

Despite these promising results, there are important limitations to acknowledge, particularly concerning the model's generalizability. While our proposed model demonstrated high predictive performance for flight delays on the specific dataset used, its generalizability to broader or unseen contexts remains an open question. Our model was trained and validated on historical flight data from U.S. domestic operations, and therefore its applicability to international flights, different regulatory environments, or airlines with distinct operational patterns may be limited. In machine learning, ensuring generalization beyond the training distribution is critical for real-world deployment. Recent work by Asif et al. [31] introduced the Advanced Zero-Shot Learning (AZSL) framework to address generalization challenges in federated learning by leveraging synthetic data and Zero-Shot Learning (ZSL) techniques. Their approach demonstrated that models could effectively adapt to unseen classes and non-IID data distributions, enhancing model flexibility and robustness across heterogeneous environments. Inspired by this, future extensions of our study could explore the integration of synthetic data generation, domain adaptation techniques, or zero-shot learning approaches to improve the model's transferability to different airports, countries, or operational conditions. This would help ensure that the flight delay prediction models remain reliable under a variety of real-world scenarios.

6. Conclusion

This study conducted a comprehensive evaluation of six machine learning classifiers for flight delay prediction under class imbalance. Performance enhancement factors, including feature engineering, feature selection, and data sampling techniques, were applied. The models were evaluated using a benchmark dataset, and their performance was assessed using various metrics.

The results of our analysis revealed that Random Forest with Random Oversampling and Decision Tree with SMOTE outperformed the other models, achieving an accuracy of 0.90 and an F1-score of 0.91. SVC also demonstrated strong performance, exhibiting balanced performance across the metrics. Overall, the study highlights the importance of careful model selection, resampling strategy, and cross-validation to achieve reliable flight delay predictions. The findings offer actionable insights for aviation stakeholders and data scientists, supporting improved operational planning, resource allocation, and decision-making. Future investigations should consider ensemble methods, domain adaptation, and deep learning frameworks to further elevate predictive accuracy while maintaining computational efficiency.

Author contributions

Conceptualization: Sarah Ahmed A Albassam, Samira Dhafir N. AlShahrani.

Formal analysis: Sarah Ahmed A Albassam, Samira Dhafir N. AlShahrani.

Methodology: Samira Dhafir N. AlShahrani.

Software: Samira Dhafir N. AlShahrani.

Supervision: Sarah Ahmed A Albassam.

Writing – original draft: Samira Dhafir N. AlShahrani.

Writing – review & editing: Sarah Ahmed A Albassam.

References

1. Wensveen J. Air transportation: a global management perspective. 9th ed. London: Routledge. 2023.
2. Nguyen C-V. Air Transport Resilience, Tourism and Its Impact on Economic Growth. Economies. 2024;12(9):236. https://doi.org/10.3390/economies12090236
3. International Air Transport Association. IATA's annual review 2024. Montreal: IATA. 2024. https://www.iata.org/en/publications/annual-review/
4. Song C, Ma X, Ardizzone C, Zhuang J. The adverse impact of flight delays on passenger satisfaction: An innovative prediction model utilizing wide & deep learning. Journal of Air Transport Management. 2024;114:102511. https://doi.org/10.1016/j.jairtraman.2023.102511
5. Kalliguddi AM, Leboulluec AK. Predictive Modeling of Aircraft Flight Delay. ujm. 2017;5(10):485–91. https://doi.org/10.13189/ujm.2017.051003
6. Xu Y, Liu L, Gao X, Zeng FF. Analysis of Factors in Flight Delay. In: Proceedings of the 2019 2nd International Conference on Mathematics, Modeling and Simulation Technologies and Applications (MMSTA 2019), 2019. https://doi.org/10.2991/mmsta-19.2019.36
7. Meel P, Singhal M, Tanwar M, Saini N. Predicting Flight Delays with Error Calculation using Machine Learned Classifiers. In: 2020 7th International Conference on Signal Processing and Integrated Networks (SPIN), 2020. 71–6. https://doi.org/10.1109/spin48934.2020.9071159
8. Atlioğlu Mc, Bolat M, Şahin M, Tunali V, Kilinç D. Supervised Learning Approaches to Flight Delay Prediction. Sakarya University Journal of Science. 2020;24(6):1223–31. https://doi.org/10.16984/saufenbilder.710107
9. Tijil Y, Dwivedi N, Srivastava SK, Ranjan A. Flight Delay Prediction Using Machine Learning Techniques. In: 2024 IEEE International Conference on Computing, Power and Communication Technologies (IC2PCT), 2024. 1909–13. https://doi.org/10.1109/ic2pct60090.2024.10486482
10. Chen J, Li M. Chained Predictions of Flight Delay Using Machine Learning. In: AIAA Scitech 2019 Forum, 2019. https://doi.org/10.2514/6.2019-1661
11. Qu J, Wu S, Zhang J. Flight Delay Propagation Prediction Based on Deep Learning. Mathematics. 2023;11(3):494. https://doi.org/10.3390/math11030494
12. Guvercin M, Ferhatosmanoglu N, Gedik B. Forecasting Flight Delays Using Clustered Models Based on Airport Networks. IEEE Trans Intell Transport Syst. 2021;22(5):3179–89. https://doi.org/10.1109/tits.2020.2990960
13. Ayaydin A, Akcayol Ma. Deep Learning Based Forecasting of Delay on Flights. Bilişim Teknolojileri Dergisi. 2022;15(3):239–49. https://doi.org/10.17671/gazibtd.1060646
14. Bisandu DB, Moulitsas I, Filippone S. Social ski driver conditional autoregressive-based deep learning classifier for flight delay prediction. Neural Comput & Applic. 2022;34(11):8777–802. https://doi.org/10.1007/s00521-022-06898-y
15. Zhou H, Li W, Jiang Z, Cai F, Xue Y. Flight Departure Time Prediction Based on Deep Learning. Aerospace. 2022;9(7):394. https://doi.org/10.3390/aerospace9070394
16. Cai K, Li Y, Fang Y-P, Zhu Y. A Deep Learning Approach for Flight Delay Prediction Through Time-Evolving Graphs. IEEE Trans Intell Transport Syst. 2022;23(8):11397–407. https://doi.org/10.1109/tits.2021.3103502

17. Li Q, Guan X, Liu J. A CNN-LSTM framework for flight delay prediction. Expert Systems with Applications. 2023;227:120287. https://doi.org/10.1016/j.eswa.2023.120287
18. Deng W, Li K, Zhao H. A Flight Arrival Time Prediction Method Based on Cluster Clustering-Based Modular With Deep Neural Network. IEEE Trans Intell Transport Syst. 2024;25(6):6238–47. https://doi.org/10.1109/tits.2023.3338251
19. Kim S, Park E. Prediction of flight departure delays caused by weather conditions adopting data-driven approaches. J Big Data. 2024;11(1). https://doi.org/10.1186/s40537-023-00867-5
20. Yuan Y, Wang Y, Lai CS. Multi-Attribute Data-Driven Flight Departure Delay Prediction for Airport System Using Deep Learning Method. Aerospace. 2025;12(3):246. https://doi.org/10.3390/aerospace12030246
21. Kaggle. Flight status prediction dataset. https://www.kaggle.com/datasets/robikscube/flight-delay-dataset-20182022. Accessed 2024 December 24.
22. Verdonck T, Baesens B, Óskarsdóttir M, vanden Broucke S. Special issue on feature engineering editorial. Mach Learn. 2021;113(7):3917–28. https://doi.org/10.1007/s10994-021-06042-2
23. Theng D, Bhoyar KK. Feature selection techniques for machine learning: a survey of more than two decades of research. Knowl Inf Syst. 2023;66(3):1575–637. https://doi.org/10.1007/s10115-023-02010-5
24. Kraiem MS, Sánchez-Hernández F, Moreno-García MN. Selecting the Suitable Resampling Strategy for Imbalanced Data Classification Regarding Dataset Properties. An Approach Based on Association Models. Applied Sciences. 2021;11(18):8546. https://doi.org/10.3390/app11188546
25. Sharma V. A Study on Data Scaling Methods for Machine Learning. INJGAADMIN, ftjijgasr. 2022;1(1). https://doi.org/10.55938/ijgasr.v1i1.4
26. Ahsan M, Mahmud M, Saha P, Gupta K, Siddique Z. Effect of Data Scaling Methods on Machine Learning Algorithms and Model Performance. Technologies. 2021;9(3):52. https://doi.org/10.3390/technologies9030052
27. Zaki MJ, Meira W Jr. Data mining and machine learning: fundamental concepts and algorithms. 2nd ed. Cambridge: Cambridge University Press; 2020.
28. Suthaharan S. Decision Tree Learning. Integrated Series in Information Systems. Springer US. 2016. p. 237–69. https://doi.org/10.1007/978-1-4899-7641-3_10
29. Rainio O, Teuho J, Klén R. Evaluation metrics and statistical tests for machine learning. Sci Rep. 2024;14(1):6086. https://doi.org/10.1038/s41598-024-56706-x PMID: 38480847
30. Alasmari R, Alhogail AA. Protecting Smart-Home IoT Devices From MQTT Attacks: An Empirical Study of ML-Based IDS. IEEE Access. 2024;12:25993–6004. https://doi.org/10.1109/access.2024.3367113
31. Asif M, Naz S, Ali F, Alabrah A, Salam A, Amin F, et al. Advanced Zero-Shot Learning (AZSL) Framework for Secure Model Generalization in Federated Learning. IEEE Access. 2024;12:184393–407. https://doi.org/10.1109/access.2024.3510756
